# Chromosome-level genome assembly of the northern Pacific seastar *Asterias amurensis*

**DOI:** 10.1038/s41597-023-02688-w

**Published:** 2023-11-04

**Authors:** Yanlin Wang, Yixin Wang, Yujia Yang, Gang Ni, Yulong Li, Muyan Chen

**Affiliations:** 1 The Key Laboratory of Mariculture, Ministry of Education, Ocean University of China, Qingdao, 266003 China; 2 CAS Key Laboratory of Marine Ecology and Environmental Sciences, Institute of Oceanology, Chinese Academy of Sciences, Qingdao, 266071 China

**Keywords:** Genomics, Molecular biology

## Abstract

*Asterias amurensis* has attracted widespread concern because of its population outbreaks, which has impacted fisheries and aquaculture, as well as disrupting local ecosystems. A high-quality reference genome is necessary to better investigate mechanisms of outbreak and adaptive changes. Combining PacBio HiFi and Hi-C sequencing data, we generated a chromosome-level *A. amurensis* genome with a size of 491.53 Mb. The contig N50 and scaffold N50 were 8.05 and 23.75 Mb, respectively. The result of BUSCO analysis revealed a completeness score of 98.85%. A total of 16,531 protein-coding genes were predicted in the genome, of which 94.63% were functionally annotated. The high-quality genome assembly resulting from this study will provide a valuable genetic resource for future research on the mechanism of population outbreaks and invasion ecology.

## Background & Summary

*Asterias amurensis* (class: Asteroidea), also known as the northern Pacific seastar, is widely distributed in the northwest and northeast Pacific, native to the coast of Alaska^1^, China^2^, Japan^3^, Korea^4^, and Russia^5^. As a benthic echinoderm with distinct evolutionary classification^6^, its reproduction mode includes not only dioecious but also asexual reproduction by arm regeneration^7,8^. Females have high fecundity and can annually spawn ~20 million eggs^3^. The planktonic stage of larva can last for seven weeks or several months, which enables them to rapidly spread in a suitable environment^9,10^. *A. amurensis* is located at the highest trophic level among the benthic invertebrates as a voracious and efficient generalist predator^11^, which has been reported to impact a variety of infaunal taxa, especially commercial bivalves^12–14^. And it has even been associated with the decline of some fish species^15^.

In the early 1980s, free-spawning starfish *A. amurensis* were first spotted in southeast Tasmania of Australia, possibly introduced from central Japan through ship ballast water^3^. Since their first detection, this starfish has successfully established populations in a short period and gradually expanded to Victoria^16–18^. As one of the most successful invasive species, *A. amurensis* became a significant threat to native assemblages, marine commercial species, and has damaged native ecosystems in Australia^13,19^. Thus, this starfish was listed as one of the high-priority marine pests in Australia^20^. Although its invasive range is limited in Australia^21^ so far, *A. amurensis* will likely continue to expand due to its high fecundity, wide environmental tolerance, and long larval duration^22^, even invading the Southern Ocean^23^. However, due to the lack of genomic information in *A. amurensis*, genetic changes associated with invasive lineages remain unknown^16,24^.

Periodic and massive outbreaks of *A. amurensis* populations have been reported in several countries, including Australia, China, and Japan, which have significantly impacted fishery and mariculture grounds, as well as destroyed the original ecological balance, leading to serious economic losses^25–27^. Unfortunately, no effective bio-control method has been reported for this pest up to now. To provide warning information for possible outbreaks of *A. amurensis*, early detection technologies have been developed based on targeting rRNA^28^ and the mitochondrial cytochrome c oxidase subunit I (COI) gene^21,29,30^. However, the mechanism of aggregation and outbreak is complex and unclear. Relevant studies require the support of a high-quality genome assembly, which may help to identify species-specific factors associated with aggregating starfish^31^.

In the present study, a *de novo* assembled chromosome-level *A. amurensis* genome was prepared using PacBio HiFi and Hi-C sequencing data. The final genome size was 491.53 Mb with scaffold N50 of 23.75 Mb. Using three approaches for gene structure annotation, we identified a total of 16,531 protein-coding genes, of which 15,643 genes were functionally annotated with at least one public database. A high-quality reference genome for *A. amurensis* will be a useful genomic resource to explore both the mechanism of population outbreak and the genetic basis underlying adaptive change during the invasion process. Meanwhile, the *A. amurensis* genome will be a noteworthy addition to the existing suite of Asteroidea genomes for future cell, developmental and evolutionary biology research.

## Methods

### Sample collection

All samples used in this study were from a male adult *A. amurensis* collected by diving in Qingdao, Shandong Province, China (36°03′04″N, 120°21′26″E) in November 2022. Fresh gonad tissue from the base of the arm was excised and washed with phosphate buffered saline (PBS, 1X). It was then immediately frozen in liquid nitrogen and transferred to −80 °C for storage. High quality DNA was extracted from gonad using DNeasy Blood & Tissue Kit (Qiagen, Germany) for long-read and short-read whole genome sequencing. To aid in structural annotation, nine tissues including gonad, body wall, madreporite, spine, mouth, stomach, muscle, podia, and eye spot were used for transcriptome sequencing. All tissues were isolated separately with scissors and forceps, and then treated in the same way as the gonad collection. Total RNA was extracted using the TRIzol reagent (Vazyme, China).

### Sequencing

For long-read sequencing, high molecular weight genomic DNA (gDNA) was fragmented to approximately 15 kb to construct a PacBio HiFi library. The sequencing library was generated using the SMRTbell Express Template Prep kit 2.0 (Pacific Biosciences, USA), following the manufacturer’s recommendations, as described in the previous study^32^. The library was finally sequenced with circular consensus sequencing (CCS) mode on the PacBio Sequel II system using a single 8 M cell. After filtering out the low-quality reads and sequence adapters, a total of 11.15 Gb CCS data were obtained with a mean length of 12.51 kb (Table 1).

**Table 1:** Statistical analysis of sequencing reads from BGI, Illumina and PacBio.

Libraries	Insert size (bp)	Clean data (Gb)	Reads number	Read length (bp)	Sequence coverage (X)
BGI reads	350	112.58	377,205,535	150	229.04
PacBio reads	15,000	11.15	890,929	12,511 (mean)	22.68
RNA-seq	350	13.47	45,079,838	150	—
Total	—	137.20	423,176,302	—	251.72

For short-read whole genome sequencing, gDNA was fragmented into approximately 350 bp for library construction. The library was sequenced on DNBSEQ-T7 platform to generate 150 bp paired-end (PE150) reads. After filtering out low-quality reads including reads shorter than 100 bp, reads that contained >10% “N”, and reads that contained >50% low-quality bases (Phred score ≤10), the clean data generated was 112.58 Gb, which covered ~229X of the genome (Table 1).

The chromosome conformation capture (Hi-C) technique was employed to assemble a chromosome-level genome. The fresh gonad was crosslinked using formaldehyde solution and digested with four-cutter restriction enzyme (DpnII). The ends of the restriction fragments were labeled with biotinylated nucleotides, and then the ligated DNA was sheared into fragments from 300 bp to 700 bp in length for Hi-C library construction. The resulting library was quantified with the Q-PCR method and sequenced with the DNBSEQ-T7 platform. After removing adapters and low-quality short reads, a total of 102.75 Gb (209.04 × coverage) of clean data was generated, with Q20 = 97.32% and Q30 = 92.33% (Table 2).

**Table 2:** Statistical analysis of sequencing data from Hi-C.

Type	Data
Raw paired reads	350,304,882
Raw Base(bp)	105,091,464,600
Clean Base(bp)	102,748,760,698
Effective Rate(%)	99.77
Q20(%)	97.32
Q30(%)	92.33
GC Content(%)	39.18

For transcriptome sequencing, total RNA of nine tissues from the same starfish was extracted and equally pooled for cDNA library construction. The resulting library was constructed by NEBNext® Ultra™ RNA Library Prep Kit (NEB, USA) according to the manufacturer’s instructions and sequenced on Illumina NovaSeq6000 system, finally generating 13.47 Gb clean data to help genome structure annotation.

### Genome assembly

Based on PaciBio HiFi reads, Hifiasm (v0.18.4)^33^ was applied for *de novo* assembly of primary contigs with default parameters. Haplotypic and heterozygous duplication was removed using purge_dups (v1.2.6)^34^ with the parameter of cutoffs ‘-l 5 -m 18 -u 54’. A primary assembly was generated, consisting of 90 contigs spanning 491.50 Mb. N50 and the maximum contig length were 8.05 and 28.59 Mb, respectively (Table 3).

**Table 3:** Assembly statistics of *A. amurensis* genome.

Type	Contig (bp)	Scaffold (bp)
Total Number	90	22
Total Length	491,503,102	491,537,102
Average Length	5,461,145	22,342,596
Max Length	28,598,918	38,009,675
N50 Length	8,054,564	23,750,475
N50 Number	19	9
N90 Length	3,441,115	15,767,093
N90 Number	54	19

**Fig. 1:**
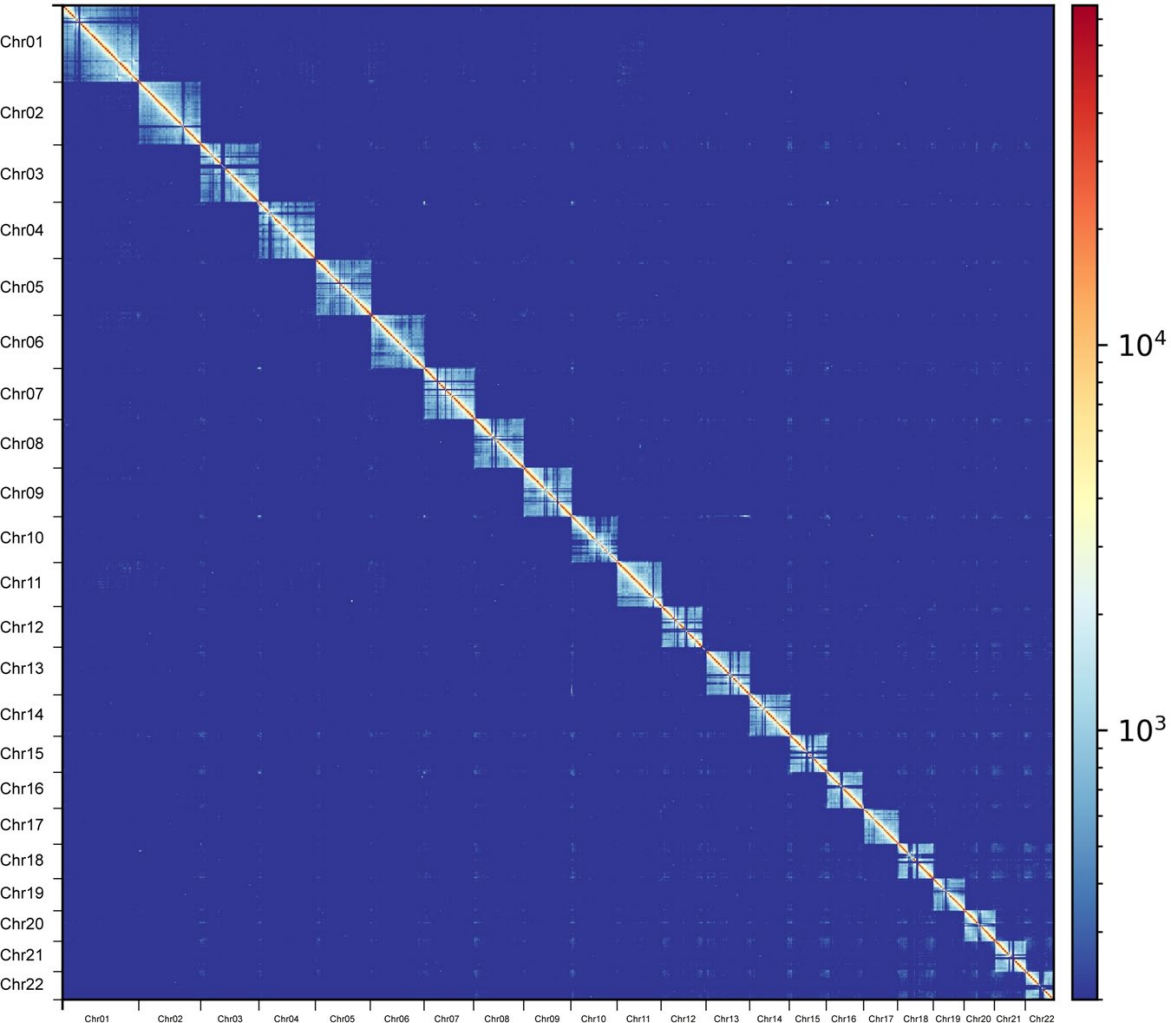
Genome-wide heatmap of Hi-C interactions among 22 chromosomes in *A. amurensis*. The scale bar represents the interaction frequency of Hi-C links.

**Fig. 2:**
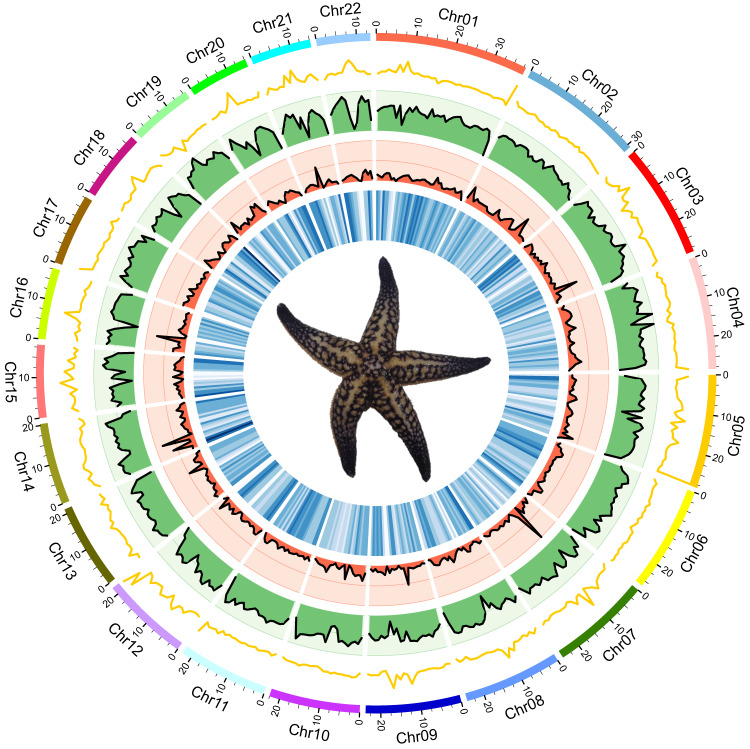
Circos plot of genomic features in *A. amurensis* genome. The tracks from outside to inside indicate: (1) length of 22 chromosomes (Mb), (2) distribution of GC content with a window of 1 Mb, (3) distribution of repeat elements with a window of 1 Mb, (4) distribution of ncRNAs with a window of 1 Mb, and (5) distribution of protein-coding genes with a window of 1 Mb.

We further scaffolded the contigs using Hi-C sequencing data to obtain a high-quality chromosome-scale genome. Juicer (v1.6)^35^ was applied for raw sequence data analysis and then 3D-DNA (v190716)^36^ was used to anchor contigs into chromosomes. The assembly was further corrected manually according to the Hi-C heatmap using JuiceboxGUI (v1.11.08)^37^, a visualization system for Hi-C contact maps. The final genome consisted of 22 chromosomes with lengths ranging from 13.43 to 38.00 Mb, and the N50 was 23.75 Mb (Table 3, Fig. 1, Fig. 2). Previous karyotype analysis^38^ of *A. amurensis* indicated that it had a diploid chromosome number of 44, which was consistent with our results.

### Annotation of repetitive elements

The Extensive *de novo* TE Annotator (EDTA, v2.0.0)^39^ and RepeatModeler (v2.0.3)^40^ were utilized to build repetitive sequence libraries for *A. amurensis* genome. We combined these two libraries as a final comprehensive repeat library for repeat annotation. Then, RepeatMasker (v4.1.2)^41^ was used to predict and classify repetitive elements of *A. amurensis* genome. Overall, sequences constituting 48.69% of the assembled genome were identified as repeats, of which the most abundant repetitive element was long terminal repeats (LTR, 19.63%), followed by DNA transposons (18.20%) (Table 4, Fig. 2).

**Table 4:** Classification of repetitive sequences in *A. amurensis* genome.

Type			Count	Length (bp)	% of Genome
Dispersed repeats	DNA transposons		747,789	89,432,495	18.20
	Retroelements	LTR	597,681	96,479,749	19.63
		LINE	2,928	1,233,195	0.25
		DIRS	416	206,153	0.04
		Penelope	2,072	791,234	0.16
	Unclassified		118,869	45,699,346	9.30
Tandem repeats	Simple repeats		82,117	4,897,016	1.00
	Low complexity		11,639	592,691	0.12
Total			1,563,511	239,331,879	48.69

### Noncoding RNA (ncRNA) annotation

Ribosomal RNAs (rRNAs) and transfer RNAs (tRNAs) were predicted by Barrnap (v0.9, https://github.com/tseemann/barrnap) and tRNAscan-SE (v2.0.11)^42^ with default parameters, respectively. Based on an alignment with Rfam database (v14.8)^43^, Infernal (v1.1.4)^44^ was used to annotate other ncRNAs, including small nuclear RNAs (snRNAs) and microRNAs (miRNAs). In total, we identified 37 miRNAs, 14,926 tRNAs, 415 rRNAs, and 202 snRNAs in *A. amurensis* genome (Table 5, Fig. 2).

**Table 5:** Classification of ncRNAs in *A. amurensis* genome.

Type		Copy number	Average length(bp)	Total length(bp)	% of genome
miRNA		37	85.62	3,168	0.0006445
tRNA		14,926	72.28	1,078,878	0.2194907
rRNA	28 S	42	2,241.43	94,140	0.0191522
	18 S	22	1,813.00	39,886	0.0081145
	5.8 S	22	117.00	2,574	0.0005237
	5 S	329	99.19	32,633	0.0066390
snRNA	CD-box	54	98.24	5,305	0.0010793
	HACA-box	27	171.41	4,628	0.0009415
	scaRNA	1	94	94	0.0000191
	splicing	120	142.28	17,073	0.0034734

### Gene prediction and functional annotation

**Fig. 3:**
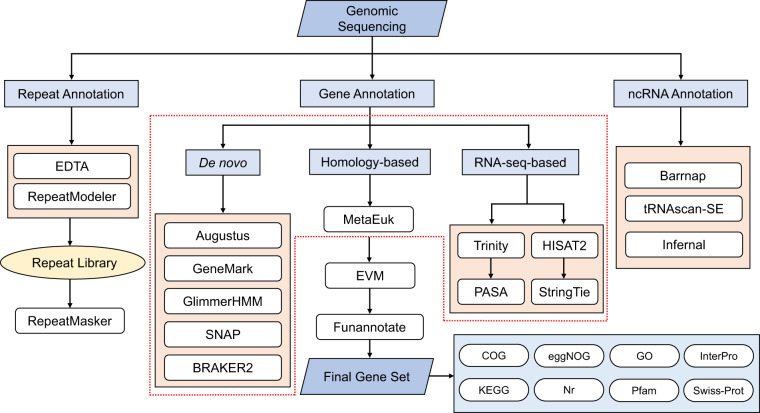
The general annotation pipeline of repetitive elements, ncRNAs, and protein-coding genes.

**Fig. 4:**
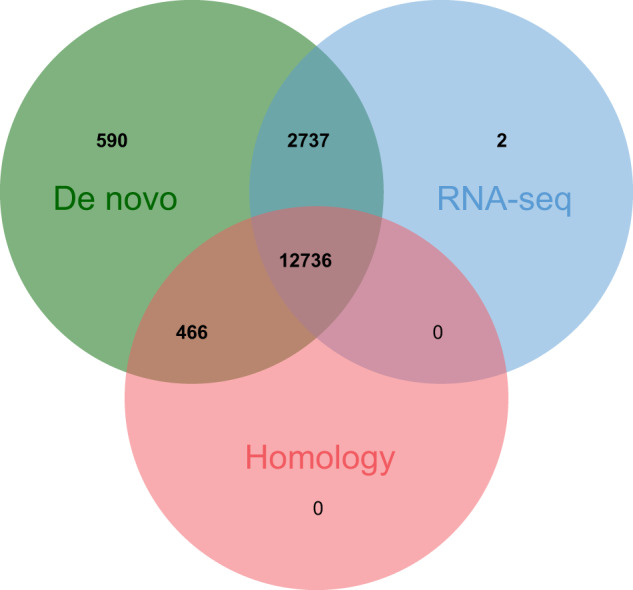
Venn diagram of gene structure prediction from *de novo*, homology-based and RNA-seq-based strategies.

**Fig. 5:**
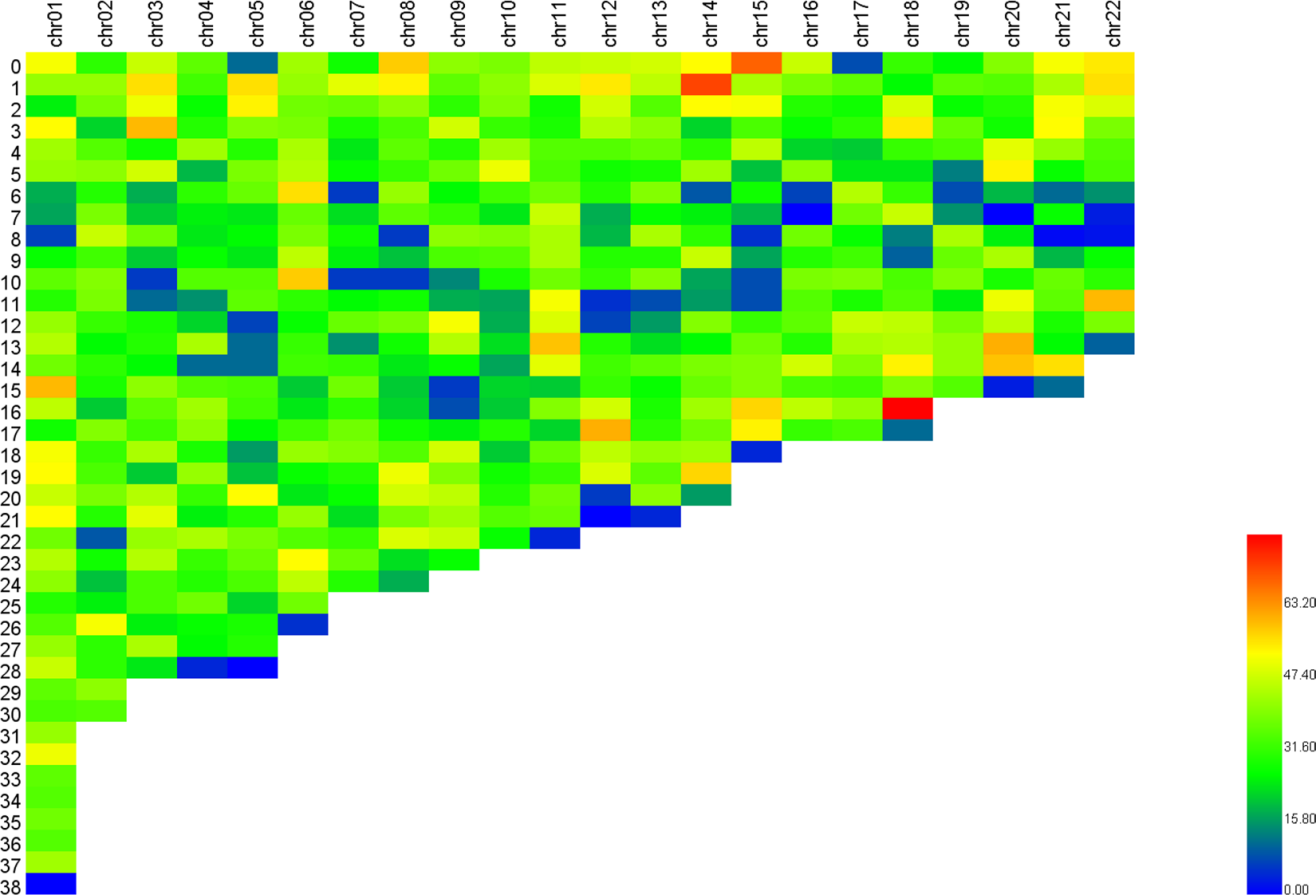
Number of genes on 22 chromosomes with a window of 1 Mb. The scale bar represents the density of genes.

**Fig. 6:**
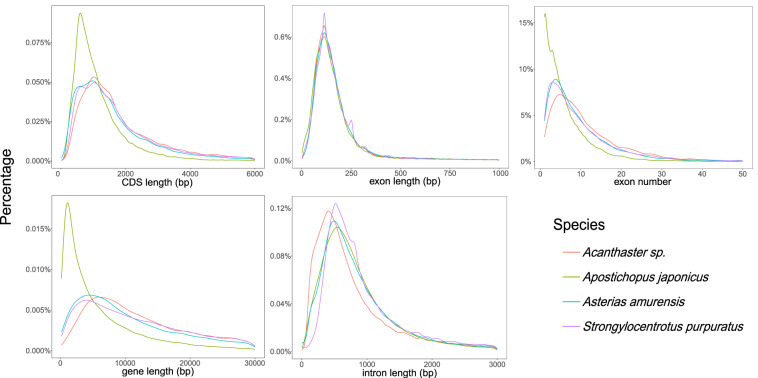
Comparisons of CDS length, exon length, exon number per gene, gene length and intron length among *A. amurensis* and other relative species.

We used three approaches for predictions of gene structures, including *de novo*, homology-based, and RNA-seq-based prediction. Augustus (v3.4.0)^45^, GlimmerHMM (v3.0.4)^46^, GeneMark (v4.69)^47^, SNAP (version 2006-07-28)^48^, and BRAKER2 (v2.1.6)^49^ were utilized for *de novo* gene model prediction and they were performed with default parameters. For homology-based prediction, we downloaded protein sequences of the crown-of-thorns starfish *Acanthaster sp*. (https://ftp.ncbi.nlm.nih.gov/genomes/all/GCF/001/949/145/GCF_001949145.1_OKI-Apl_1.0/), sea urchin *Strongylocentrotus purpuratus* (https://ftp.ncbi.nlm.nih.gov/genomes/all/GCF/000/002/235/GCF_000002235.5_Spur_5.0/), and sea cucumber *Apostichopus japonicus* (https://ftp.ncbi.nlm.nih.gov/genomes/genbank/invertebrate/Apostichopus_japonicus/latest_assembly_versions/GCA_002754855.1_ASM275485v1/) from National Center for Biotechnology Information (NCBI) as references and used MetaEuk (version aa7ac2eb7334405ad57d50d78361e3dcd61bb27a)^50^ with default parameters to predict gene structures. For RNA-seq-based prediction, we firstly mapped short RNA reads to reference genome using HISAT2 (v2.2.1)^51^ with the parameter ‘-dta’ and then assembled transcripts using StringTie (v2.2.1)^52^. Meanwhile, the Program to Assemble Spliced Alignments (PASA, v2.4.1) pipeline (https://github.com/PASApipeline/PASApipeline) was used to identify possible coding regions based on *de novo* transcriptome assembled by Trinity (v2.14.0)^53^ with default parameters. Then, EvidenceModeler (EVM, v1.1.1)^54^ and Funannotate (v1.8.14) pipeline (https://github.com/nextgenusfs/funannotate) were applied for combining predicted results from three strategies and removal of low-quality gene annotations. Based on the RNA-seq data of *A. amurensis* from this study, adult stomach tissue^55^, and bipinnaria larval^16^ from other studies, PASA (v2.4.1) was applied for the update of untranslated regions (UTRs). The general annotation pipeline applied in the present study was shown in Fig. 3. As a result, a total of 16,531 protein-coding genes were predicted and the average gene length was 17,803.19 bp, with an average coding sequence (CDS) length of 1,885.87 bp and average exon number of 10.07 (Table 6). Among them, 12,736 (77.04%) genes were supported by evidence from all three strategies (Fig. 4). We also counted the density of genes on different chromosomes with a window of 1 Mb in length (Fig. 5) and simply compared gene length, CDS length, exon length, intron length and exon number per gene of *A. amurensis* and other species used in homology-based predictions (Fig. 6). The 1 Mb region with the largest number of annotated genes were from the end of chromosome 18 (Fig. 5).

**Table 6:** Statistical results of the gene structure annotation in *A. amurensis* genome.

	Gene set	Gene Number	Gene length (bp)	CDS length (bp)	Average intron length (bp)	Average exon length (bp)	Exon per gene
De novo	Augustus	20,742	12,558.23	1,743.97	1,466.01	208.19	8.38
	GlimmerHMM	59,965	7,146.75	847.98	2,106.12	197.82	4.29
	GeneMark	22,312	9,008.46	1,587.86	1,172.18	216.61	7.33
	SNAP	25,901	26,508.15	2,030.64	2,200.07	167.47	12.13
	BRAKER2	24,353	10,549.42	1,702.42	1,848.07	206.99	8.22
RNA-seq	PASA	14,250	14,259.92	1,315.87	1,970.86	298.97	7.15
	HISAT2 & StringTie	15,047	21,787.79	1,922.51	2,005.33	383.69	12.26
Homology	*A. planci*	10,063	11,124.46	1,309.15	2,680.73	282.37	4.66
	*A. japonicus*	6,200	5,562.53	956.09	2,715.86	355.90	2.67
	*S. purpuratus*	7,265	6,095.00	1,106.24	2,621.27	382.42	2.90
Final		16,531	17,803.19	1,885.87	1,743.54	283.00	10.07

**Fig. 7:**
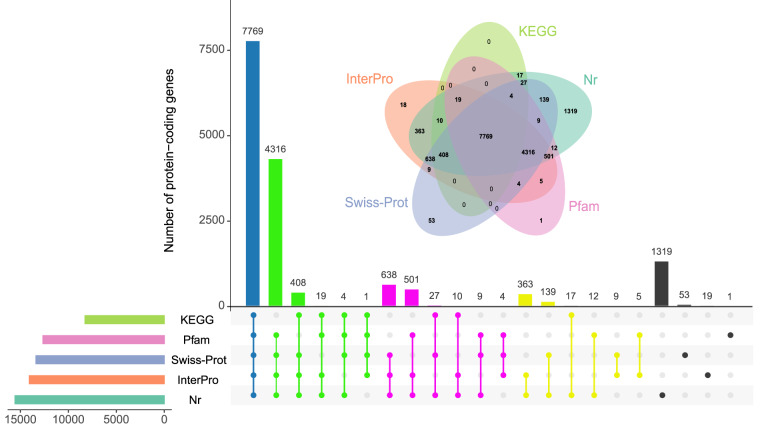
Upset plot and Venn diagram of functional annotation for protein-coding genes based on different databases, including InterPro, KEGG, Nr, Pfam, and Swiss-Prot.

Functional annotations were accomplished using Funannotate pipeline, based on databases including Clusters of Orthologous Groups of Proteins (COG)^56^, eggNOG^57^, Gene Ontology (GO)^58^, Interpro^59^, Kyoto Encyclopedia of Genes and Genomes (KEGG)^60^, NCBI non-redundant protein (Nr), Pfam^61^, and Swiss-Prot^62^. The results showed that 15,643 protein sequences (94.63%) were annotated with at least one public database (Table 7, Fig. 7).

**Table 7:** Summary of the functional gene annotation in *A. amurensis* genome.

Database	Number	Percent(%)
Total	16,531	—
COG	13,657	82.61
EggNOG	14,245	86.17
GO	10,373	62.75
InterPro	14,060	85.05
KEGG	8,254	49.93
Pfam	12,640	76.46
Swiss-Prot	13,376	80.91
Nr	15,551	94.07
Annotated	156,43	94.63
Unannotated	888	5.37

### Comparative genomic analysis

**Fig. 8:**
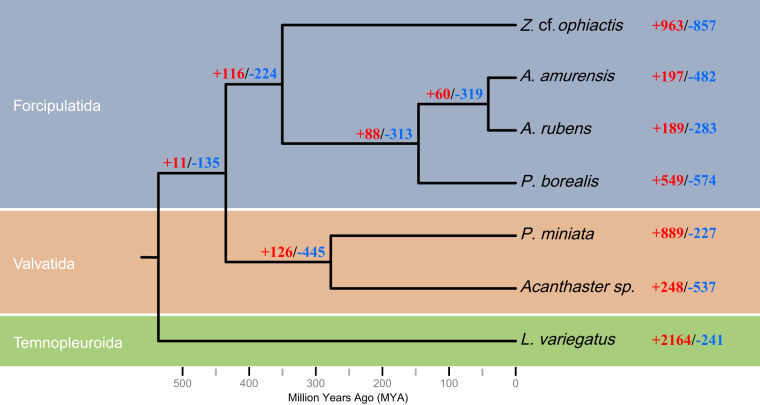
Phylogenetic and gene family evolution analysis between *A. amurensis* and the other five asteroid species. The sea urchin *L. variegatus* was selected as an outgroup. All species were colored according to different orders. The scale below represents the divergence time. The number of expanded (+red) and contracted (-blue) gene families were shown alongside the species.

The longest protein sequences of *A. amurensis* and other five asteroid species including *Acanthaster sp*.^63^, *Asterias rubens*^64^, *Patiria miniata*^65^, *Plazaster borealis*^66^, and *Zoroaster* cf. *ophiactis*^67^ were utilized to identify orthologous groups using OrthoFinder (v2.5.5)^68^ with the parameters ‘-S diamond’, and the sea urchin *Lytechinus variegatus*^69^ was selected as an outgroup. A total of 5,315 single-copy orthogroups were obtained for subsequent phylogenetic analysis. Based on multiple sequence alignments of the single-copy orthogroups using MAFFT (v7.520)^70^, IQ-TREE (v2.2.3)^71^ was applied for construction of the species trees with the parameters ‘-m MFP -bb 1000’ and the best model of GTR + F + I + R4. Predictably, *A. amurensis* was most closely related to *A. rubens* and *P. borealis* from the family Asteriidae (Fig. 8). Then, divergence times were estimated using MCMCTREE in PAML (v4.9i)^72^ based on the divergence time (*A. amurensis* vs *L. variegatus*: 461.1.5-600.0 million years ago) extracted from TIMETREE (http://www.timetree.org/). The expansion and contraction of gene families were analyzed by Computational Analysis of gene Family Evolution (CAFE, v5.0.0)^73^ with a p-value of 0.05. The results revealed that 197 and 482 gene families were expanded and contracted in *A. amurensis*, respectively (Fig. 8).

## Data Records

The PacBio, BGI, RNA-seq, and Hi-C sequencing data have been deposited in the NCBI Sequence Read Archive (SRA) database under the accession numbers of SRR24902114^74^, SRR24831139^75^, SRR24871501^76^, and SRR24835318^77^. The final chromosome assembly has been deposited in GenBank with assembly accession number GCA_032118995.1^78^. The genome annotation files are available in the Figshare database^79^.

## Technical Validation

### Nucleic acid quality

The concentration and quality of DNA were evaluated using Nanodrop 2000 spectrophotometer (Thermo Fisher Scientific, USA) and agarose gel electrophoresis, respectively. RNA integrity was assessed using Agilent 2100 Bioanalyzer (Agilent Technologies, USA).

### Genome assembly and annotation quality evaluation

The quality of the final chromosome-level genome assembly was assessed using four methods as follows. Firstly, we mapped clean PE150 reads from whole genome sequencing to *A. amurensis* genome using BWA-MEM (v0.7.17)^80^ and calculated the mapping rate using samtools (v1.9)^81^, resulting in a genome coverage rate of 99.95% and a mapping rate of 99.61%. Secondly, the results of Benchmarking Universal Single-Copy Orthologs (BUSCO, v5.2.2)^82^ analysis based on 954 genes of metazoa_odb10 database indicated that 951 (99.69%) core metazoan genes were detected in *A. amurensis* genome, consisting of 943 (98.85%) complete and 8 (0.84%) fragmented genes (Table 8). Thirdly, the Core Eukaryotic Genes Mapping Approach (CEGMA, v2.5)^83^ based on 248 core eukaryotic genes showed that 236 (95.16%) genes were identified in the final genome assembly. Finally, meryl (v1.3)^84^ was used to generate k-mer counts based on paired-end reads generated by whole genome sequencing, and Merqury (v1.3)^84^ was utilized to estimate the consensus quality value (QV) of *A. amurensis* genome, resulting in a QV of 48.51. The results from the four methods above revealed the high accuracy and completeness of the final genome assembly.

**Table 8:** BUSCO evaluation of gene annotation in *A. amurensis* genome.

Type	Percentage
Complete BUSCOs (C)	98.85% (943)
Complete and single-copy BUSCOs (S)	97.80% (933)
Complete and duplicated BUSCOs (D)	1.05% (10)
Fragmented BUSCOs (F)	0.84% (8)
Missing BUSCOs (M)	0.31% (3)
Total	100% (954)

## Data Availability

No custom code was utilized in this study. Data processing was performed by relevant pipelines and software according to the manual and protocols and the version as well as useful parameters have been described in the Methods section. The default parameters as developers suggested were used in those pipelines and software of which parameters were not specifically mentioned in this work.
